# Converting the E. coli Isochorismatase Nicotinamidase into γ-Lactamase

**DOI:** 10.1128/spectrum.00985-21

**Published:** 2022-02-16

**Authors:** Xiaoyan Guo, Licao Chang, Haibo Jin, Junge Zhu, Yong Tao, Sheng Wu, Jianjun Wang

**Affiliations:** a College of New Materials and Chemical Engineering, Beijing Institute of Petrochemical Technology, Beijing, People’s Republic of China; b State Key Laboratory of Microbial Resources, Institute of Microbiology, Chinese Academy of Sciences, Beijing, People’s Republic of China; c Beijing Key Laboratory of Fuels Cleaning and Advanced Catalytic Emission Reduction Technology, Beijing, People’s Republic of China; University of Hong Kong

**Keywords:** γ-lactamase, isochorismatase superfamily, substrate entrance tunnel

## Abstract

Nicotinamidase (Nic) (E.C.3.5.1.19) is a representative protein of the isochorismatase superfamily from Escherichia coli. Despite showing no (+) γ-lactamase activity, its active site constellations (ASCs) are very similar to those of two other known (+) γ-lactamases (Mhpg and RutB), indicating that it could be a latent (+) γ-lactamase. In this study, the primary sequences of the five representative proteins of the isochorismatase superfamily from E. coli were aligned, and a “lid”-like unit of a six-residue loop (112GENPLV117) was established. The Nic protein was converted to a (+) γ-lactamase by eliminating the loop. A conversion mechanism was proposed in which a more compact binding pocket is formed after lid deletion. In addition, the “shrunk” binding pocket stabilized the small substrate and the catalysis intermediate, which triggered catalysis. Moreover, we identified another latent (+) γ-lactamase in the E. coli isochorismatase superfamily and successfully converted it into an active (+) γ-lactamase. In summary, the isochorismatase superfamily is potentially a good candidate for obtaining novel (+) γ-lactamases.

**IMPORTANCE** γ-Lactamases are important enzymatic catalysts in preparing optically pure γ-lactam enantiomers, which are high-value chiral intermediates. Different studies have presumed that the isochorismatase superfamily is a candidate to obtain novel (+) γ-lactamases. By engineering its substrate entrance tunnel, Nic, a representative protein of the isochorismatase superfamily, is converted to a (+) γ-lactamase. Tunnel engineering has proven effective in enhancing enzyme promiscuity. Therefore, the latent or active γ-lactamase activities of the isochorismatase superfamily members indicate their evolutionary path positions.

## INTRODUCTION

**FIG 1:**
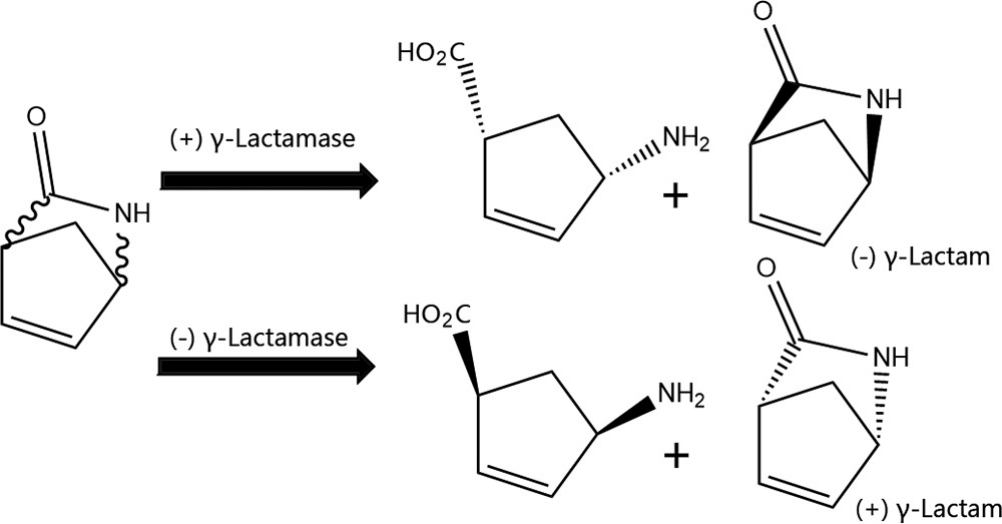
Reactions catalyzed by γ-lactamases.

A small enzyme subclassification in amidases is γ-lactamase (1) (E.C.3.5.2.X) hydrolyzes γ-lactam (2-azabicyclo[2.2.1]hept-5-en-3-one). It shows absolute enantioselectivity toward pure enantiomers of γ-lactam (Fig. 1). Optically pure γ-lactam is a crucial chiral synthon for several high-value chiral drugs (2). However, discovering new γ-lactamases with better catalytic properties (higher activity or enantioselectivity) using microbial genomes is challenging (3–7).

Previously, we discovered two novel (+) γ-lactamases, including RutB (Escherichia coli) (5) and Mhpg (Microbacterium hydrocarbonoxydans) (6), which were initially annotated as isochorismatases in GenBank. Another isochorismatase from M. hydrocarbonoxydans, showing (+) γ-lactamase promiscuity, was found in another study (3).

Enzyme promiscuity suggests a divergent evolution from a common ancestor (8). Therefore, (+) γ-lactamase activity could be a promiscuous function of the isochorismatase superfamily ancestor. Notably, promiscuity in one member of an enzyme family suggests a probability that other members are also promiscuous (8).

The E. coli isochorismatase superfamily comprises five families, including nicotinamidase (E.C.3.5.1.19), nicotinamidase-related protein, *N*-carbamoylsarcosine amidohydrolase (E.C.3.5.1.59), isochorismatase (E.C. 3.3.2.1), and a family of unknown function (9). In this study, Nic is the representative protein of the nicotinamidase family and RutB is categorized as the *N*-carbamoylsarcosine amidohydrolase (6, 10).

Genes from E. coli are usually easy to handle and express in the same hosts. Therefore, this study presumed that the E. coli isochorismatase superfamily is an excellent candidate to obtain novel (+) γ-lactamases like RutB. The active site constellations (ASCs) of RutB and Mhpg were compared with those of the other four representative proteins from the E. coli isochorismatase superfamily. Here, Nic showed ASCs very similar to those of RutB and was selected for enzyme conversion.

We compared the primary sequence among the five representative proteins of the isochorismatase superfamily, and a unique loop structure was established in the Nic protein. The 0.6 kDa loop was a highly flexible peptide comprising six amino acid residues (GENPLV). The loop acted as a “lid” that blocked the substrate entrance tunnel. Subsequently, the Nic protein was converted into an active (+) γ-lactamase by eliminating the lid.

## RESULTS

### Enzyme expression and purification and determination of γ-lactamase activity.

**FIG 2:**
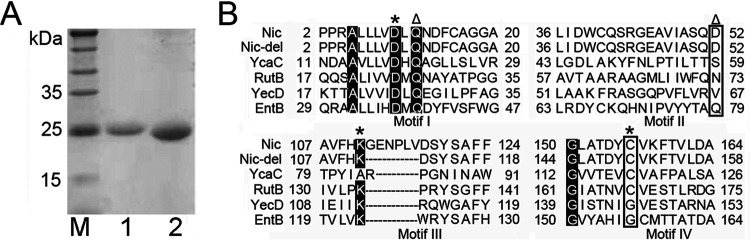
SDS-PAGE of the purified proteins (A) and multiple alignments of proteins from E. coli isochorismatase superfamily (B). (A) M, SDS-PAGE protein marker; 1, Nic; 2, Nic-del. Around 20 μg of each protein was loaded. (B) There are four motifs among the five proteins. Residues in motif I, motif III, and motif IV constituted the residues of the catalytic triads (residues marked using an asterisk). Motif I and motif II constitute the two substrate-stabilizing residues (marked using triangles). Abbreviations: Nic, nicotinamidase from E. coli (accession number NP_416282); YcaC, E. coli YcaC (accession number AAC73983); RutB, RutB from E. coli (accession number WP_001393558.1); YecD, isochorismatase from E. coli (accession number WP_022887063); and EntB, isochorismatase from E. coli (accession number AAC73696).

The lengths of the *nic* and *nic-del* structural genes were 642 bp and 624 bp, respectively. Both genes were expressed as fusion proteins with His tag at carboxyl (C) termini (pBAD MCS). The recombinant enzymes were expressed in the soluble fraction of the bacterial lysate. The purified proteins were subjected to SDS-PAGE to evaluate their purity after single-step purification on the Ni-chelating columns (Fig. 2A). Liquid chromatography-mass spectrometry (LCMS) subsequently revealed the relative monomer molecular masses of Nic and Nic-del to be 24.2 kDa (Fig. S1, supplemental material) and 23.6 kDa (Fig. S2, supplemental material), respectively. After they were tested for their (+) γ-lactamase activity, we observed that (+) γ-lactamase activity was present only in Nic-del.

### Identifying a lid-like loop in Nic through sequence alignment.

Apart from Nic and RutB, the isochorismatase superfamily of E. coli has three other representative proteins (9). They include YecD (a nicotinamidase-related protein), EntB (isochorismatase), and YcaC (a protein family of unknown function). Among these five proteins (EntB, RutB, Nic, YcaC, and YecD), YecD and EntB had the Asp-Lys-Gly triad, whereas the other proteins had the Asp-Lys-Cys catalytic triad. Subsequently, these five proteins were compared by sequence alignment, and four motifs among them were identified (Fig. 2B). Three motifs were relatively conserved (motif I, motif III, and motif IV). They also constituted the residues of the catalytic triads (residues marked using an asterisk, Fig. 2B). On the other hand, motif I and motif II constituted the two substrate-stabilizing residues (denoted by triangles, Fig. 2B). Lastly, in motif III, Nic had a six-residue insertion (112GENPLV117) (Fig. 2B), unlike other members.

### Identifying catalytic residues using an ASC structure alignment.

Previously, we found two isochorismatases that showed (+) γ-lactamase activities (5, 6). They included Mhpg (Microbacterium hydrocarbonoxydans) and RutB (E. coli). They possessed a presumed catalytic triad comprising Asp-Lys-Cys and two residues (Gln and His/Asn) involved in stabilizing substrates. The catalytic triad and the stabilizing residues (Gln and His/Asn) constituted an ASC (Table 1). These five residues are crucial in maintaining the (+) γ-lactamase activities. This is because they were nearly abolished when these residues were substituted with alanines (data not shown).

**TABLE 1:** Constituents of the ASCs

Enzyme	Catalytic residues	Stabilizing residues
RutB	Asp25-Lys134-Cys167	Gln27, Asn73
Mhpg	Asp9-Lys84-Cys118	Gln11, His51
Nic	Asp10-Lys111-Cys156	Gln12, Asp52

**FIG 3:**
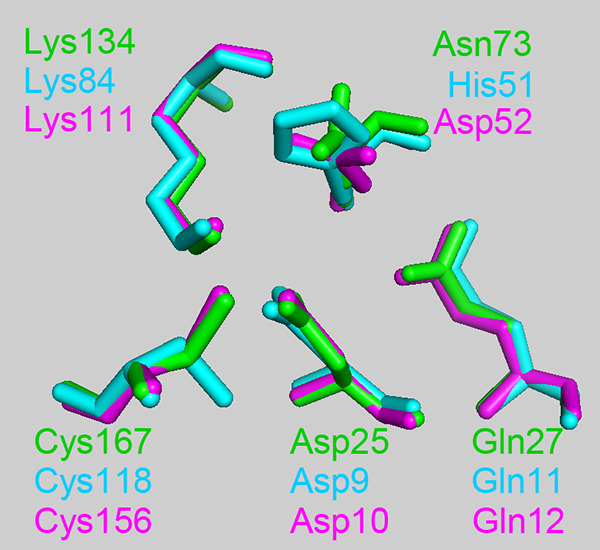
Superimposition of the ASCs. Homology modeling was conducted using the SWISS-MODEL server. RutB (green) template is 3IRV, a cysteine hydrolase from Pseudomonas syringae pv. *phaseolicola* 1448A; the Mhpg (cyan) template is 3MCW, a putative hydrolase of the isochorismatase superfamily from Chromobacterium violaceum ATCC 12472; the Nic (purple) template is 2WTA, which is a nicotinamidase from Acinetobacter baumannii. Structure alignment was done using the PyMOL software.

The spatial structures of Mhpg, RutB, and Nic were superimposed to visualize structural similarities and differences of these structures, and it was clear that Nic had ASCs similar to those of the other structures (Fig. 3). Furthermore, the root mean square deviation (RMSD) values of ASCs between the three enzymes and the RutB (3IRV) homology model template were calculated. Subsequently, the ASCs of the other three representative proteins from the E. coli isochorismatases superfamily were compared to those of 3IRV (RMSD to 3IRV was 0.16, 0.87, and 1.62 Å for YecD, EntB, and YcaC, respectively). Notably, RutB and Nic had ASCs very similar to those of 3IRV, and the two ASCs showed small RMSD values (RMSD to 3IRV was 0.06, 0.29, and 0.19 Å for RutB, Mhpg, and Nic, respectively) (Fig. 3). However, Nic is an inactive γ-lactamase.

### Converting Nic into (+) γ-lactamases.

**FIG 4:**
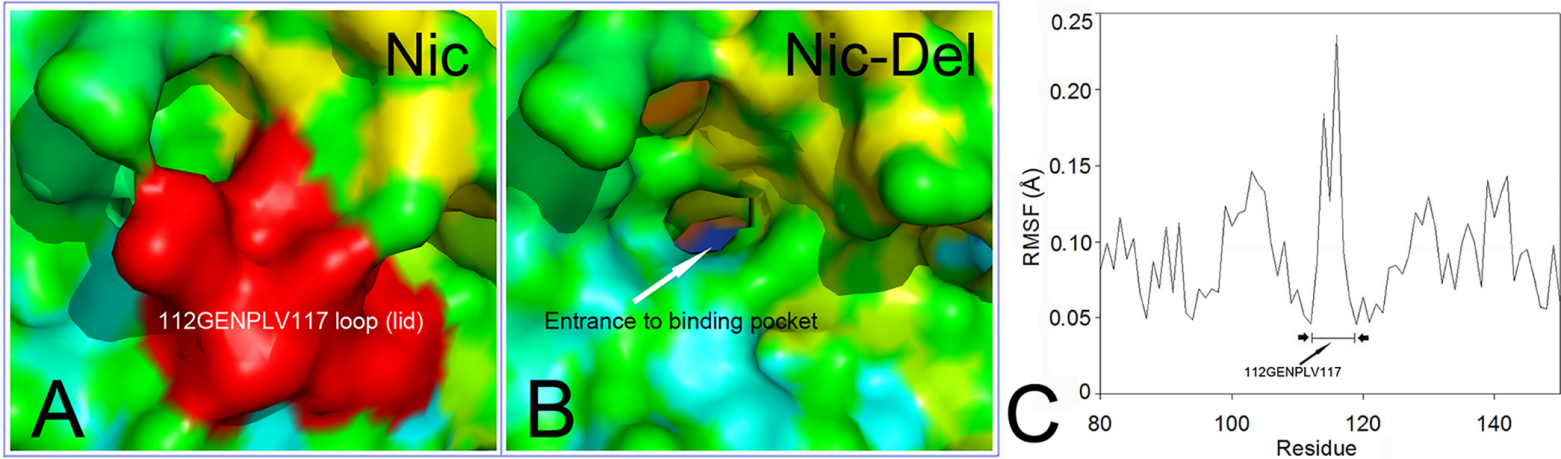
The lid structure of Nic (A), Nic-del protein without the lid (B), and RMSF for Nic-del residues in molecular simulation (C). (A and B) Nic and Nic-del proteins are displayed in surface mode (in cyan, green, and yellow), whereas the lid (112GENPLV117) is displayed in red. (C) The residue average RMSF for Nic-del in MD is 0.096 Å.

The six-residue fragment (112GENPLV117) formed a lid-like loop in front of the Nic binding pocket. The lid is a very flexible structure that possibly blocks substrates from the binding pocket (Fig. 4A). We speculated that eliminating the loop could facilitate substrate diffusion through the substrate entrance tunnel into the binding pocket (Fig. 4B). A molecular dynamic (MD) simulation of the Nic protein revealed that the six-residue loop was more flexible than that of the nearby structures in MD. In addition, these residues showed a sharp peak on the root mean square fluctuation (RMSF) plot (Fig. 4C). RMSF value is a parameter displaying the motion and flexibility of protein residues (11).

Then, the lid was deleted following standard methods provided by the QuikChange technique, and the Nic-del mutant was tested for (+) γ-lactamase activity. The Nic-del mutant protein had a (+) γ-lactamase activity (*K*_m _= 123 mM, *k*_cat _= 80 min^−1^) much lower than that of RutB (5) (*K*_m _= 30 mM, *k*_cat_ = 1.0 × 10^6^ min^−1^).

Nic had an ASC with Asp10-Lys111-Cys156 and Gln12-Asp52 (Table 1). According to the RMSD comparison with Nic-del, the ASC made no great deviations (RMSD = 0.1 Å). The residue numbers of one of the five catalytic residues were altered (from Cys156 to Cys150). These findings confirmed that the lid is a flexible structure that contributes little to ASC rigidity. Moreover, these five ASC residues were also mutated to alanines, and all the resulting mutants were inactive or almost inactive toward (+) γ-lactam (data not shown).

### Molecular docking.

**FIG 5:**
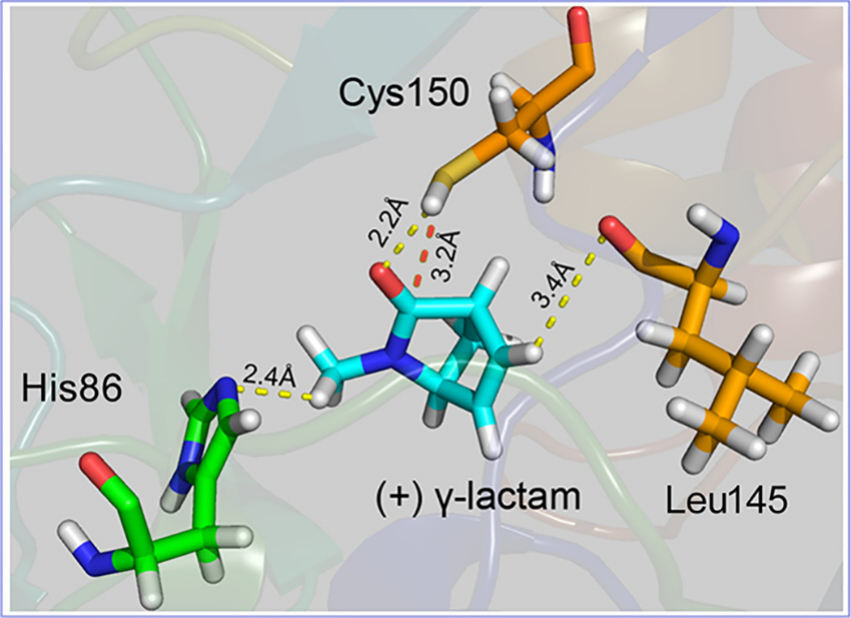
Docking (+) γ-lactam into the binding pocket of Nic-del. Hydrogen bonds are in yellow dashes. The nucleophilic attack distance (between the sulfur atom of Cys150 and C6 of the γ-lactam) is in the red dash.

Two enantiomers of γ-lactam molecules were docked into the Nic-del binding pocket, and as per the docking analysis results (data not shown), only the (+) γ-lactam molecules showed a rational pose. Furthermore, three hydrogen bonds were formed during the (+) γ-lactam docking process. They existed between NE2 (nitrogen atom) of His86 and H18 (hydrogen atom) of (+) γ-lactam, HG (hydrogen atom) of Cys150 and O7 (oxygen atom) of (+) γ-lactam, and O (oxygen atom) of Leu145 and H16 (hydrogen atom) of (+) γ-lactam (Fig. 5). The hydrogen bonds stabilized the substrate to initiate the Cys150 catalysis. However, the (−) γ-lactam molecule was not docked into the binding pocket (no hydrogen bond formed), indicating that Nic-del is a (+) γ-lactamase with absolute enantioselectivity for (+) γ-lactam. The (+) γ-lactam molecule was docked into the Nic binding pocket, with formation of only two hydrogen bonds (HG of Cys156 and O7 of [+] γ-lactam, O of Leu145 and H16 of [+] γ-lactam). This docking result indicated insufficient stabilization of the unlike in the Nic-del binding pocket.

### Mechanism for the (+) γ-lactamase conversion for Nic-del.

**FIG 6:**
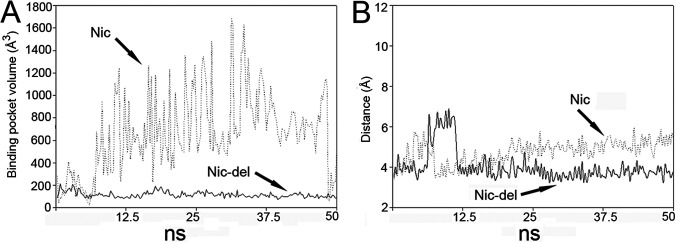
Monitoring the binding pocket volume (BPV) (A) and nucleophilic attack distance (B) in molecular simulation. Data for Nic are represented by dotted lines, while data for Nic-del are represented by solid lines. Volumes of binding pocket were calculated using the POVME 3.0 software. The nucleophilic attack distance is between the sulfur atom of Cys150 and C6 of the γ-lactam.

The binding pocket volume (BPV) analyzed through the POVME software during molecular dynamic (MD) simulation of Nic-del protein showed that the BPV of Nic-del was steadily maintained at different volumes from around 150 Å^3^ to 200 Å^3^. In contrast, the BPV of native Nic protein was larger and drastically changed during MD (Fig. 6A). As a member of the isochorismatase superfamily, Nic catalyzed the isochorismic acid hydrolysis to produce pyruvic acid and 2,3-dihydro-2,3-dihydroxybenzoate (12). The volumes of the compounds involved in its catalysis were larger than those in γ-lactamase catalysis. Moreover, Nic showed capacious binding pockets to accommodate these substrates. Compared to Nic, small substrates (γ-lactam) in large binding pockets demonstrated that reaction intermediates were not quickly stabilized during catalysis. In contrast, compact binding pockets provided a conducive environment for small substrate catalysis, and this was the same in Nic-del. During MD, more hydrogen bonds were formed between γ-lactam and Nic-del (3 at most, Fig. 5) than between γ-lactam and Nic (2 at most), confirming the above assumption. Furthermore, based on the γ-lactam hydrolysis mechanism, thiolate, facilitating nucleophilic attack at the carbonyl group of γ-lactam, initiated catalysis (3). During MD, the distance between the sulfur atom (Cys150) and C6 (γ-lactam) was maintained at around 3.5 Å (Fig. 6B), which triggers catalysis. Lastly, as demonstrated in Fig. 6B, during MD, the distance in native Nic was beyond 5.0 Å.

## DISCUSSION

A lid or “flap” is a special and subtle structure in protein molecules located in front of the substrate tunnel or over active sites. Its structure unit is a loop or helix and is more flexible than other secondary structures, like β sheet. Additionally, it is an amphipathic structure that comprises hydrophilic and hydrophobic residues (13). The lid structure exists widely in the lipase superfamily, e.g., porcine pancreatic lipase (14). The principal function of the lid is to regulate activity control by switching the active site conformation. Also, it contributes to enzyme-substrate selectivity and thermostability (14). Of note, lids were also found in other proteins like adenylate kinase (15), acyltransferase (16), and chaperones (17).

To some extent, the lid is a part of the substrate entrance tunnel or is a device before the substrate entrance tunnel. Therefore, lid engineering is the engineering of the substrate entrance tunnel. Many studies have proved that engineering a substrate entrance tunnel is an effective method to enhance enzyme promiscuity (18–20). This study provided another example to demonstrate the efficiency of this method. Nic is an inactive (+) γ-lactamase due to blockade of the entrance tunnel using a lid loop. Nevertheless, by eliminating the loop, Nic-del was converted to an active (+) γ-lactamase.

Taken together, Nic, a representative protein of the E. coli isochorismatase superfamily, is a latent (+) γ-lactamase. This is because it displays active site constellations (ASCs) very similar to those of the other two known (+) γ-lactamases. In this study, Nic was converted to a (+) γ-lactamase by eliminating a loop. To date, our group has identified a native (RutB) and a latent (Nic) (+) γ-lactamase in the E. coli isochorismatase superfamily. Recently, we converted YecD to an active γ-lactamase through single residue replacement (manuscript in preparation). Therefore, the isochorismatase superfamily is probably an excellent candidate to obtain novel (+) γ-lactamases. We believe that EntB and YcaC are also potential latent γ-lactamases; hence, it is imperative to explore them further.

From an evolutionary point of view, RutB could have been the intermediate during evolution, whereas YecD, Nic, and other members could have reached the “final destination” as specific isochorismatases. In a nutshell, RutB is an isochorismatase with (+) γ-lactamase promiscuity, whereas YecD and Nic are isochorismatases with latent (+) γ-lactamase activities that were recovered by either replacement of a glycine residue or deletion of a loop insertion.

## MATERIALS AND METHODS

### Chemicals, bacterial strains, plasmids, and culture media.

Racemic Vince lactam was purchased from Acros Organics (Beijing, PRC). All the other chemicals used were analytical reagents.

E. coli strain K-12 substrain MG1655 (conserved in ATCC, strain collection number ATCC 47076) cultures were grown in either lysogeny broth (LB) medium or on LB agar plates at 37°C. The E. coli strain TOP10 (TransGen Biotech, Beijing, PRC) cells were used for cloning and protein expression studies. The plasmid pBAD/MCS (Invitrogen, CA, USA) was used for standard cloning and expression in E. coli cells. Lastly, a 50 μg/mL ampicillin concentration was added to the medium to isolate bacterial strains carrying the appropriate recombinant plasmids.

### Extraction and purification of DNA.

The chromosomal DNA isolation from E. coli strain K-12 substrain MG1655 was achieved using the TIANamp bacteria kit (Tiangen, Beijing, PRC). Subsequently, plasmids were isolated using the TIANprep minikit, and DNA was purified using the TIANgel midi kit. All the above procedures were performed following the specified protocols from the manufacturer.

### Gene cloning and expression in E. coli.

The *nic* gene was amplified from the genomic DNA of E. coli strain K-12 substrain MG1655 using the nic F and nic R primer pair. The primer sequences are outlined as follows: nic F, 5′-GGGAATTCCATATGCCCCCTCGCGCCCTGTTA-3′; nic R, 5′-CCCAAGCTTCCCCTGTGTCTCTTCCCAGTCTG-3′.

The PCR cycling protocol comprised an initial denaturation step at 95°C for 5 min, followed by 30 cycles at 95°C for 1 min, 53°C for 1 min, and 72°C for 1.5 min and a final elongation step at 72°C for 10 min. PCR was performed using the Red-*Pfu* DNA polymerase (Biocolors, Beijing, PRC). The PCR products were cloned into the NdeI and HindIII sites of the pBAD/MCS plasmid to generate pBAD*nic*.

The construct was transformed into E. coli TOP10 cells and grown overnight in LB broth at 37°C. Then, 5 mL of the overnight culture was transferred into 500 mL of fresh LB broth and cultured under the same conditions until the optical density at 580 nm (OD_580_) value reached 0.8. Arabinose was then added to obtain a final concentration of 1.0 mM, and the cells were incubated for 12 h. Finally, the cells were harvested through centrifugation (4,000 × *g* for 10 min).

### Purification of His-tagged proteins on Ni-chelating columns.

We performed purification using the Novagen (New Jersey, USA) buffer protocol with some minor modifications as previously described in our study (5). Finally, the fractions containing the target protein were collected and dialyzed against 5,000 mL of 20 mM phosphate buffer (pH 7.2) for desalting. Notably, the buffer was changed thrice over a period of 24 h.

### Homology modeling.

Nic, RutB, and Mhpg structures were obtained via homology modeling using the SWISS-MODEL Homology Modeling server (https://swissmodel.expasy.org/). The templates are listed in Table 2.

**TABLE 2:** Templates employed during homology modeling

Protein	Template	Template information
RutB	3IRV	A cysteine hydrolase from Pseudomonas syringae
Mhpg	3MCW	A hydrolase from Chromobacterium violaceum
Nic	2WTA	A nicotinamidase from Acinetobacter baumannii

### Building of the ASCs.

Enzymes from the isochorismatase superfamily have an Asp-Lys-Cys/Gly catalytic triad and two residues involved in substrate stabilization (Gln and His/Asp/Asn) (12). These five residues constitute the catalytic residues, and the pattern of the spatial organization of the catalytic residues is the active site constellation (ASC). In our study, the ASC-corresponding residues for related enzymes were inferred through sequence alignment (Table 1). Ultimately, the ASC structure alignments and RMSD calculations were performed using PyMOL software (21).

### Construction of Nic-del mutant.

Nic-del was generated through QuikChange PCR (22) using primers P1 and P2, whose sequences are outlined as follows: P1, Nic-del upstream, 5′-GGTGTTCCATAAAAGTTACAGTGCCTTTTT-3′; P2, Nic-del downstream, 5′-AAAAAGGCACTGTAACTTTTATGGAACACC-3′.

The PCR mix was performed in a 25 μL final volume containing 10× KOD buffer (2.5 μL), MgCl_2_ (1 μL, 25 mM), deoxynucleoside triphosphate (dNTP; 5 μL, 2 mM each), primers (0.5 μL of each pair of primers, 2.5 μM), template plasmid (pBADnic, 1 μL, 10 ng/μL), and 1 unit of KOD DNA polymerase. The PCR protocol comprised two rounds of amplification cycles (5 cycles in the first round and 25 cycles in the second round) with an initial denaturation at 94°C for 10 min. The first round of the PCR program consisted of five cycles of 40 s of denaturation, 40 s of annealing at 53°C, and 50 s of elongation at 68°C. The second round consisted of 25 cycles of 1 min of denaturation, 1 min of annealing at 60°C, and 6 min of elongation at 68°C. The final elongation step was for 30 min at 72°C. Subsequently, the template plasmid was removed from the 10 μL reaction mix by digesting it with 1 unit of DpnI (New England Biolabs, Beijing, PRC). Finally, the mixture was transformed into E. coli TOP10 and mutations in the clones were confirmed through DNA sequencing.

### Molecular docking.

Molecular docking was conducted using AutoDock Vina software version 1.1.2. This software combines knowledge-based potentials and empirical scoring (23). The binding site parameters and the docking box dimensions were determined by docking the ligand in the catalytic cavity (local docking). The docking results were analyzed based on the binding structure criteria, binding energy, and possible interactions between ligand and the key protein residues.

### MD simulations.

The GROMACS software was used for the MD simulations that targeted the apo-protein and protein-ligand complexes (24). The protein-ligand complex was placed in a simulation box that was a 12-surface polyhedron and contained a water solvent (TIP3P model). Ions (Na^+^and Cl^−^) were added to attain overall system neutrality. Energy minimization was executed to eliminate the unfavorable system interaction contacts. The system was then balanced under the isothermal-isobaric conditions. Berendsen thermostat and Parrinello–Rahman barostat models were used to maintain a temperature of 300 K and pressure of 1 bar, and the systems were simulated for 50 ns each. Notably, the result was recorded for every 0.01 ns. Root mean square fluctuation (RMSF) and distance between the sulfur atom of Cys150 and C6 of the γ-lactam ring were calculated using the GROMACS software and represented as a chart using Sigmaplot. Finally, the binding pocket volumes were calculated using the POVME version 3.0 software (25).

### Standard reaction conditions and γ-lactamase activity assay.

Racemic γ-lactam was used as the substrate in γ-lactamase activity assays. The activity was assayed by adding 40 μg of the enzyme to 200 μL of the 4.6 mM substrate solution (pH 8.0; 50 mM Tris-HCl buffer), and the reaction solution was incubated at 35°C for 5 min. After transformation, the reaction solution was extracted with 100 μL ethyl acetate and analyzed using chiral high-performance liquid chromatography (HPLC). The conversion rate, yield, and enantiomeric excess (*ee*) of the isolated γ-lactam enantiomer were determined through HPLC (Waters, MA, USA) equipped with a Daicel CHIRALPAK AS-H column (250 by 4 mm; Daicel Chemical Industries, Tokyo, Japan). The mobile phase, a mixture of acetonitrile and isopropyl alcohol at a volume ratio of 90:10, was used at a flow rate of 1.0 mL/min. The remaining γ-lactam was detected by measuring absorbance at 230 nm using a UV-visible (UV-Vis) detector (486 series; Waters). The retention times of benzamide, (+) γ-lactam, and (−) γ-lactam were 4.9, 6.5, and 7.9 min, respectively. Notably, the *ee* value was defined as 100 × (A − B)/(A + B), where A and B are the amounts of two γ-lactam enantiomers. Lastly, one enzyme activity unit (U) was defined as the amount of enzyme that catalyzes the conversion of one substrate nanomole per minute.

### Protein concentration and SDS-PAGE determination.

Protein concentration was determined using the bicinchoninic acid (BCA) protein assay kit (Thermo Fisher Scientific, Beijing, PRC) per the instructions provided by the manufacturer and using bovine serum albumin (BSA) as a standard.

SDS-PAGE was performed as described previously (26) with a 6% polyacrylamide stacking gel and a 12% polyacrylamide separating gel modification. Lastly, protein purity was estimated using Glyko BandScan software (Glyko, Novato, USA).

### Determination of the protein’s molecular weight using LCMS.

The proteins were subjected to an EASY-nLC 1000 interfaced via a Nanospray Flex ion source to an Orbitrap Fusion Tribrid mass spectrometer (Thermo Fisher Scientific, USA) (nano-liquid chromatography-tandem mass spectrometry [LC-MS/MS]) for analysis. This analysis was done at the Technological Platform of Mass Spectrum Centre of Institute of Microbiology, Chinese Academy of Sciences. The protein was loaded onto a trap column (C_18_, 3 μm particles, 100 μm inside diameter [i.d.], 3 cm length, Dr. Maisch GmbH) and separated using an analytical column (C_18_, 1.9 μm particles, 150 μm i.d., 15 cm length, Dr. Maisch GmbH) at a flow rate of 400 nL/min. The LC gradient time was 30 min and was composed of solvent A (0.1% formic acid) and solvent B (acetonitrile, 0.1% formic acid). The gradient was first 20 to 70% B for 25 min and finally 70 to 100% B for 5 min. The precursor MS1 scan (*m/z* 400 to 2,000) was acquired in the Orbitrap at a resolution setting of 120,000. The molecular weight was calculated using the deconvolution technique employing the Xtract algorithm of Xcalibur Qual Browser software (Thermo Fisher Scientific).

### Statistical analysis.

The BLASTX program (27) was used for protein homology search, and the Vector NTI version 8.0 sequence analysis program package (InforMax) was used for sequence alignments. All computer-aided modeling figures were prepared using the PyMOL software (21).
