# Supplementary material for: Converting the E. coli Isochorismatase Nicotinamidase into γ-Lactamase
Source: Microbiol Spectr. 2022 Feb 16;10(1):e00985-21. doi: 10.1128/spectrum.00985-21 (PMC8849098; doi:10.1128/spectrum.00985-21)
Supplement: SUPPLEMENTAL FILE 1 — Supplemental material. Download SPECTRUM00985-21_Supp_1_seq5.pdf, PDF file, 0.1 MB [file spectrum00985-21_supp_1_seq5.pdf]

Supplemental Material

Material and methods

Determination of the protein’s molecular weight using Liquid Chromatography Mass Spectrometry (LCMS)

The proteins were subjected to an EASY-nLC 1000 interfaced via a Nanospray Flex ion source to an Orbitrap Fusion Tribrid mass spectrometer (Thermo Fisher Scientific, USA) (nano-LC-MS/MS) for analysis. This analysis was done at the Technological Platform of Mass Spectrum Centre of Institute of Microbiology, Chinese Academy of Sciences. The protein was loaded onto a trap column (C18, 3 μm particles, 100 μm ID, 3 cm length, Dr. Maisch GmbH) and separated using an analytical column (C18, 1.9 μm particles, 150 μm ID, 15 cm length, Dr. Maisch GmbH) at a flow rate of 400 nL/min. The LC gradient time was 30 min and was composed of solvent A (0.1% formic acid) and solvent B (acetonitrile, 0.1% formic acid). The gradient was first, 20-70% B for 25 min, and finally 70-100% B for 5 min. The precursor MS1 scan (m/z 400–2000) was acquired in the Orbitrap at a resolution setting of 120,000. The molecular weight was calculated using the deconvolution technique employing the Xtract algorithm of Xcalibur Qual Browser software (Thermo Fisher Scientific).

Results

LCMS Results

LCMS revealed the relative monomer molecular masses of Nic and Nic-del to be 24.2 kDa (Fig. S-1) and 23.6 kDa (Fig. S-2), respectively.

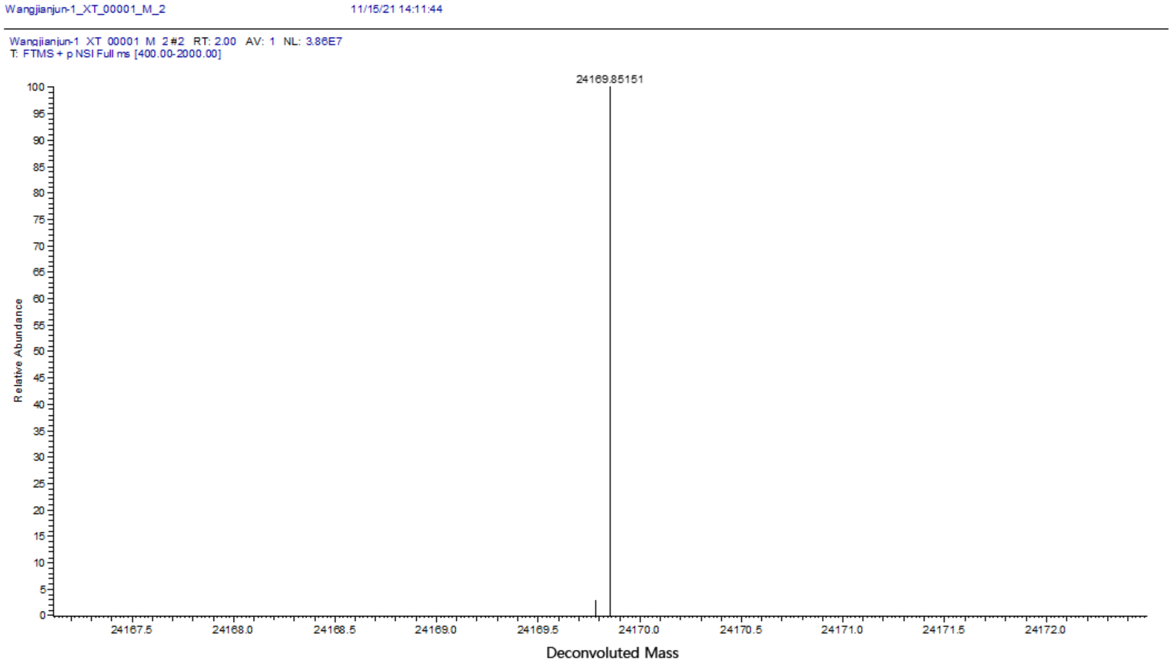

Fig. S-1 LCMS calculation result for Nic

Molecular mass of Nic; 24169 dalton

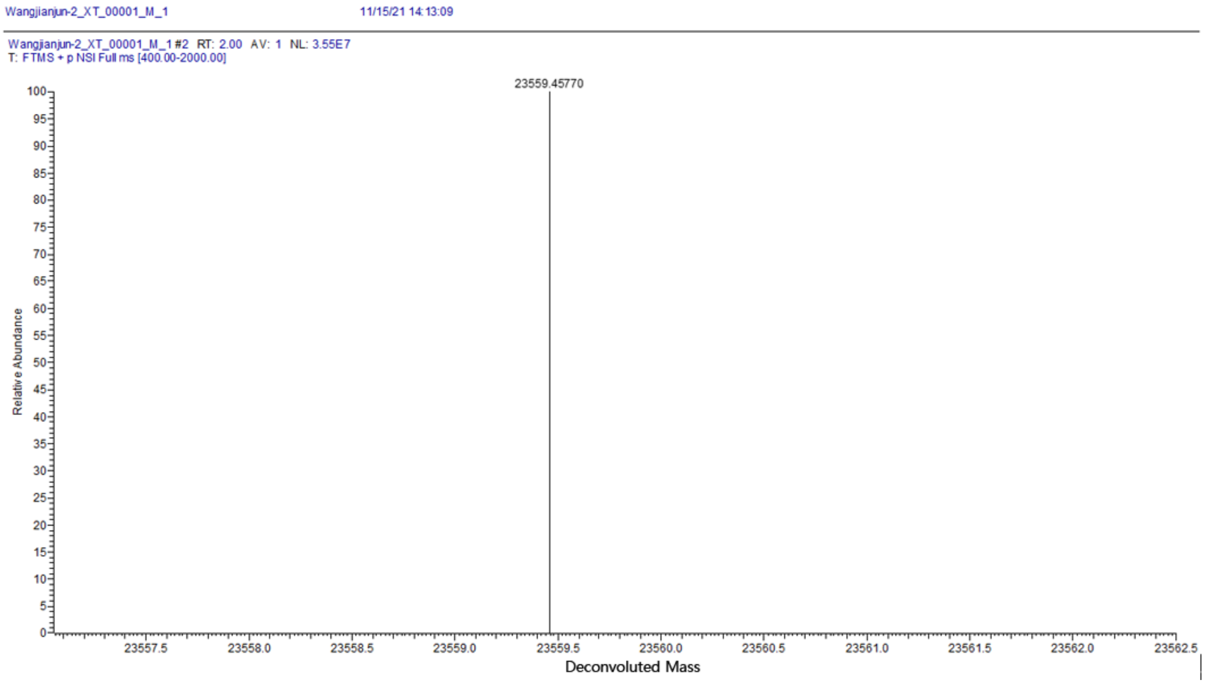

Fig. S-2 LCMS calculation result for Nicdel

Molecular mass of Nicdel; 23559 dalton
